# Evaluation of completeness and timeliness of data in the National Information System for Notifiable Diseases for spotted fever in the state of São Paulo, Brazil, 2007-2017

**DOI:** 10.1590/S2237-96222023000200011

**Published:** 2023-07-14

**Authors:** Daniele Rosa Xavier, Michellin Pereira de Albuquerque, Sílvia Von Tiesenhausen de Sousa-Carmo, Adriano Pinter

**Affiliations:** 1 Universidade de São Paulo, Programa de Pós-Graduação em Saúde Pública, São Paulo, SP, Brazil; ² Secretaria de Estado da Saúde, Centro de Informações de Vigilância Epidemiológica, São Paulo, SP, Brazil; 3 Superintendência de Controle de Endemias, São Paulo, SP, Brazil

**Keywords:** Spotted Fever, Health Information Systems, Disease Notification, Data Accuracy, Fiebre Maculosa, Sistemas de Información en Salud, Notificación de Enfermedades, Exactitud de los Datos, Febre Maculosa, Sistemas de Informação em Saúde, Notificação de Doenças, Confiabilidade dos Dados

## Abstract

**Objective:**

to evaluate the completeness and timeliness of notifications of cases of spotted fever (SF) held on the Notifiable Health Conditions Information System (SINAN) in São Paulo State, Brazil, from 2007 to 2017.

**Methods:**

this was a descriptive and ecological study of confirmed human cases of SF regarding completeness and timeliness of ten fields of the notification form (good if ≥ 90% for most variables); time series analysis was performed using the Prais-Winsten technique.

**Results:**

we analyzed 736 records; among essential fields, only “Discharge date” showed poor completeness (68.5%). Timeliness was good for the “Investigation” and “Closure” fields; other time lapses were not adequate.

**Conclusion:**

in São Paulo state, data completeness was good for most variables, whereas timeliness was not adequate (except for “Closure” and “Investigation”), pointing to the need for health education and communication actions about SF.

## Resumo

**Objetivo:**

avaliar completitude e oportunidade das notificações de casos de febre maculosa (FM) no Sistema de Informação de Agravos de Notificação (Sinan), no estado de São Paulo, no período de 2007 a 2017.

**Métodos:**

estudo descritivo e ecológico dos casos humanos confirmados de FM, mediante análise dos atributos completitude e oportunidade de dez campos da ficha de notificação (boas quando ≥ 90% para a maioria das variáveis); na análise das tendências das séries temporais, utilizou-se a técnica de Prais-Winsten.

**Resultados:**

foram analisados 736 registros; dos campos essenciais, apenas “Data da alta” demonstrou baixa completitude (68,5%); “Investigação” e “Encerramento” tiveram boa oportunidade; demais intervalos não foram adequados.

**Conclusão:**

no estado de São Paulo, a completitude dos dados foi boa na maioria das variáveis, porém não adequada quanto à oportunidade (exceto para “Encerramento” e “Investigação”), apontando para a necessidade de ações de educação e comunicação em saúde sobre FM.

## Resumen

**Objetivo:**

evaluar la completitud y oportunidad de las notificaciones de casos de fiebre manchada (FM) en el Sistema Nacional de Información sobre Enfermedades de Declaración Obligatoria (Sinan) en el estado de São Paulo, Brasil, de 2007 a 2017.

**Métodos:**

estudio descriptivo y ecológico de casos humanos confirmados de FM para completitud y oportunidad de diez campos del formulario (bueno si ≥ 90% para la mayoría de las variables). Se realizó una regresión lineal para analizar las tendencias de las series temporales.

**Resultados:**

entre los campos esenciales, solo “Fecha de alta” mostró baja completitud (68,5%). “Investigación” y “Cierre” tuvieron buena oportunidad; otros intervalos no fueron adecuados.

**Conclusión:**

en São Paulo, la completitud fue buena para la mayoría de las variables, pero no adecuada en cuanto a la oportunidad, excepto para “Cierre” e “Investigación”, lo que apunta a la necesidad de acciones de educación y comunicación en salud sobre FM.

**Table:** 

Study contributions	
**Main results**	In São Paulo state the majority of the spotted fever variables had good completeness; closure and investigation had good timeliness; and laboratory investigation timeliness was inadequate.
**Implications for services**	The study can contribute to better resource allocation in areas such as surveillance, health worker training, as well as adoption of regionalized health policies.
**Perspectives**	The situation analyses provide more information for public health authorities, including for the re-evaluation of activities commonly considered bureaucratic, with subsequent reflection in health indicators.

## INTRODUCTION

Spotted fever (SF) is an acute febrile zoonotic disease, caused by bacteria species of the *Rickettsia* genus.^1^ In São Paulo State, two distinct diseases related to SF have been identified: one caused by the *Rickettsia rickettsii* species, traditionally known as Brazilian SF, and the other caused by the *Rickettsia parkeri* bacterium, referred to as SF.^1^ *R. rickettsii* is transmitted by ticks of the *Amblyomma sculptum* and *Amblyomma aureolatum* species, while *R. parkeri* is transmitted by ticks of the *Amblyomma ovale* species.^2^ ^)-(^ ^5^

The clinical picture of SF is characterized by different levels of severity and high case fatality ratio in humans,^6^ whereby fatality can reach 80% in advanced forms if not treated.^7^ The disease is an important public health problem, which is why it has been included on the Ministry of Health list of compulsorily notifiable diseases since 2001.^8^ With effect from 2014, all SF cases, both suspected and confirmed, must be notified immediately on the Notifiable Health Conditions Information System (Sistema de Informação de Agravos de Notificação - SINAN) within 24 hours.^9^ ^),(^ ^10^

In Brazil, 2,293 SF cases were recorded between 2007 in 2020.^11^ In São Paulo State,^12^ 936 cases were notified in the last five years, 549 (58.7%) of which progressed to death. The majority of cases notified in São Paulo state in that period were male (84.8%), the highest proportion corresponded to the 20-59 age group (520 cases; 55.6%), and the case fatality ratio was estimated to be 54.4%.^12^

Production of high quality epidemiological information is both strongly recommended and highly desirable, in order for data analyses to able to represent the real magnitude and health status of an event in a given territory.^13^ Completeness and timeliness are indicators of data quality used in performance reviews, and are recommended by national^14^ and international^15^ health authorities for identifying populations and areas at epidemiological risk, as well as assisting health action programming.

Completeness is understood to mean the proportion of fields (mandatory and/or essential) filled in on data collection instruments.^13^ ^)^ Poor filling in of notification form fields leads to production of deficient and less reliable data, contributing to a poorer understanding of the dynamics of a disease, due to incorrect indicators of incidence, mortality and fatality, for example.^13^ ^),(^ ^16^ ^),(^ ^17^

Timeliness, in turn, is the time lapse between different stages of the surveillance process and refers to the time taken by an Epidemiological Surveillance Service to obtain information in a timely and efficient manner, offering input for more accurate decision making by health authorities.^18^ Analyzing “timeliness” can contribute to the improvement of epidemiological surveillance and health system information management, and to the identification of possible interfering factors related to health service users, health professionals and laboratories, such as access to health services, human resource training and sample processing time.^19^

The objective of this study was to evaluate the completeness and timeliness of data notified on the SINAN system for SF cases in São Paulo State between 2007 and 2017, with the aim of contributing to improving the SF epidemiological surveillance process; as well as to analyze SINAN quality spatial distribution regarding timeliness, in order to identify discrepancies throughout São Paulo State.

## METHODS

This was a descriptive and ecological study of confirmed human cases of SF recorded on the SINAN in São Paulo State, Brazil, between 2007 and 2017.

São Paulo state covers an area of 248,219,481 km². In 2017, it had an estimated population of 46 million inhabitants (96.0% living in urban areas), representing population density of 166.23 inhab./km².^20^ The State is comprised of 645 municipalities, 39 of which comprise the Metropolitan Region of São Paulo City.

Notification of SF cases on the SINAN began in 2007. The study selected the period from 2007 to 2017, as 2017 was the last year with available data. The database was made available by the Epidemiological Surveillance Center of the São Paulo State Department of Health on September 13, 2018, without personal data enabling identification of individuals, but with all notified and confirmed SF cases. The database was previously audited by Health Department technical staff in order to exclude any duplicate records.

We included in our analysis confirmed cases that met at least one of the following Ministry of Health criteria:^14^ ^)^

a) isolation of pathogens, via positive molecular or immunohistochemistry tests; 

b) titer seroconversion at least four times between two paired samples, 14 to 21 days apart; 

c) compatible clinical-epidemiological picture, including individuals who lived in or frequented areas subject to the presence of the vector tick, SF transmission or risk; and 

d) São Paulo State being the probable infection site and/or place of residence of the infected person.

The SINAN SF notification form has 63 fields to be filled in, classified according to the breakdown provided by the information system data dictionary: (i) mandatory fields, whereby missing information implies non-inclusion of the notification or investigation on the SINAN, and (ii) essential fields, the filling in of which is not mandatory, whereby missing information influences the calculation of epidemiological or operational indicators.

The completeness of the database was evaluated for the following essential fields: “Date of hospital admission”, “Date of discharge”, “Date of first serological sample collection” and “Date of second serological sample collection”. Among the essential fields, “Date of hospital admission” was selected as it indicates more severe cases that require hospitalization; the other fields served as indirect parameters for evaluation of the care provided to the individual, considering health surveillance guidelines. Moreover, when filling in these fields the “Unknown” option cannot be used.

With regard to timeliness, we considered the following variables and their respective time lapses:

a) time lapse between date of onset of first symptoms and notification date, timely if up to seven days;

b) time lapse between notification date and date of first serological sample collection, timely if up to 24 hours, excluding negative values;

c) time lapse between notification date and date of investigation, timely if up to seven days;

d) time lapse between notification date and date of input to the system, timely if up to 15 days;

e) time lapse between notification date and date of case closure, timely if up to 60 days;

f) time lapse between onset of first symptoms and first serological sample collection date, timely if up to seven days;

g) time lapse between first serological sample collection and date of the second serological sample collection, timely between 14 and 21 days.

Time lapses greater than 365 days and negative time lapses were excluded from our analyses of timeliness.

Percentage completeness of each variable was calculated by dividing total filled in cases that were not null (excluding ‘unknown’ cases) by total confirmed cases, for each year of the study period. The percentage of timely notifications for each variable was obtained by dividing the number of notifications that met the time limit criterion by the total number of confirmed cases with valid notifications.

According to the parameters recommended by the United States Centers for Disease Control and Prevention (CDC)^15^ and by the Brazilian Ministry of Health,^14^ the following criteria were used to classify the data as to their completeness and timeliness - good 

(≥ 90.0%), regular (≥ 70.0% to < 90.0%) or poor (< 70.0%) - for all the variables; except for case “Closure” timeliness, for which the following classification was used - good (≥ 80.0%), regular (≥ 70.0% to < 80.0%) or poor (< 70.0%).^14^ Due to the lack of established parameters in the literature about completeness and timeliness of “Serological analyses”, we opted to use the “Closure” categories-values adopted by the Ministry of Health.^14^

We prepared box-plots for each of the time lapses analyzed. The time lapses (in days) for the timeliness attribute were characterized by means of descriptive statistics (mean; standard deviation; median; minimum and maximum values). 

The time trend analyses for completeness and timeliness were performed using the Prais-Winsten technique, which enables the fit of the logistic regression model using the ordinary least squares method.^21^ First, the percentage curves found over time were visually analyzed. Prais-Winsten analysis was performed for each variable, which corrects for possible first-order autocorrelation. The time trend was considered to be rising if Annual Percentage Change (APC) was positive with a p-value < 0.05, falling if APC was negative with a p-value < 0.05, or stable when any APC had a p-value > 0.05.

We performed all statistical analysis using R software, versions 2.18.24 and 4.2.2.

São Paulo State is divided into 28 Epidemiological Surveillance Groups (Grupos de Vigilância Epidemiológica - GVE).^22^ We undertook a study of spatial distribution per municipality of notification and per GVE with the purpose of evaluating completeness and timeliness in a regionalized manner. We opted to detail the timeliness parameters for the “Notification versus date of symptom onset”, “Investigation”, “Data input” and “Closure” variables, in relation to actions carried out by municipal health services. We prepared choropleth maps to represent the timeliness percentages, using the Quantum GIS application, version 3.2.

This research was approved by the Universidade de São Paulo Public Health Faculty Research Ethics Committee: Opinion No. 2.961.082, issued on October 15, 2018, as per Certificate of Submission for Ethical Appraisal No. 97917318.0.0000.5421, with subsequent agreement of the São Paulo State Department of Health.

## RESULTS

Between 2007 and 2017, 739 cases of SF were confirmed in São Paulo state. Three cases (0.4%), who did not reside in São Paulo state, were excluded from the present analysis. As such, we analyzed 736 cases, 77.2% (568) of which were autochthonous. 

Essential field completeness was found to be good for “Date of first serological sample collection” (97.9%), “Date of hospital admission” (96.6%) and “Date of second serological sample collection” (90.3%). Completeness of the “Date of discharge” field was regular (84.4%). At least 59.5% of cases were reported in a timely manner, i.e. within seven days from symptom onset. Only 33% of cases had their first serological sample collected within 24 hours from the time of notification. More than 81% of confirmed cases (588 cases) were closed in a timely manner.

**Figure 1:**
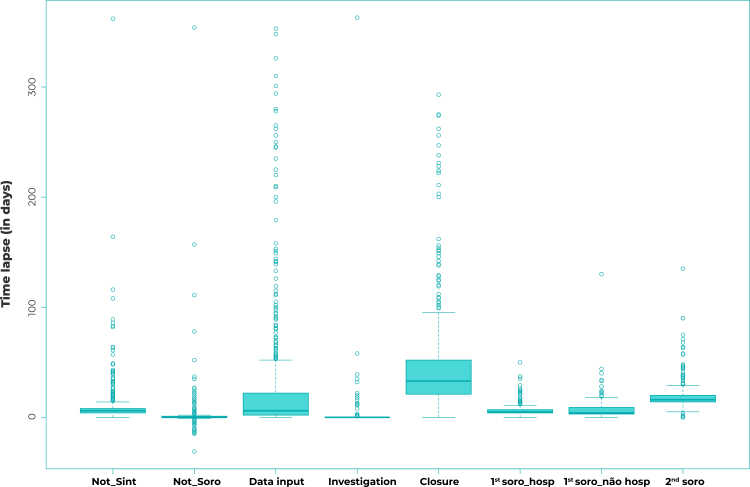
- Distribution of the time lapses (in days) of the timeliness parameters of confirmed spotted fever cases by year of notification, São Paulo State, 2007-2017

Legend: Not_Sint = notification vs. symptom onset; Not_Soro = notification vs. serological sample collection; 1^st^ soro_hosp = first serological sample in hospitalized cases; 1^st^ soro_nao hosp = first serological sample in non-hospitalized cases; 2^nd^ soro = second serological sample.

The time lapses (in days) between the onset of first symptoms, serological sample collections (1st and 2nd samples), input date, investigation, case closure, and the date of notification are shown in the box-plots in Figure 1. The mean, median, standard deviation, maximum and minimum values of the time lapses (in days) found by our timeliness analyses, by reporting year, are shown in Table 1. The greatest data dispersion was found for the “Data input” variable, which showed a six-day median and a mean ranging from 12.3 to 67.2 days. There was little data dispersion (median equal to zero: 0.0) between notification and first serological sample collection; and between notification and investigation, the means of which ranged from 1.2 to 9.3 days and from 0.0 to 9.8 days, respectively. Median time between notification and case closure was 34 days, also with little data dispersion. Little variability was also found in the notification data regarding the onset of symptoms and the collection of both serological samples.

**Table 1:** Statistical description of the time lapses (in days) of the timeliness analyses of confirmed spotted fever cases by year of notification, São Paulo State, 2007-2017

Year	Notification-Symptoms		Notification-Serology		Data input		Investigation		Closure		1^st^ serological sample collection (hospitalized cases)		1^st^ serological sample collection (non-hospitalized cases)		2^nd^ serological sample collection	
	Mean ± sd	Median (Min-Max)	Mean ± sd	Median (Min-Max)	Mean ± sd	Median (Min-Max)	Mean ± sd	Median (Min-Max)	Mean ± sd	Median (Min-Max)	Mean ± sd	Median (Min-Max)	Mean ± sd	Median (Min-Max)	Mean ± sd	Median (Min-Max)
2007	9.9 ± 10.5	7 (0;49)	1.2 ± 3.6	0 (0;16)	28.7 ± 54.9	6 (0;245)	0.6 ± 2.3	0 (0;11)	9.9 ± 10.5	42.5 (11;238)	1.2 ± 3.6	7 (0;18)	28.7 ± 54.9	7 (0;18)	0.6 ± 2.3	19.5 (0;18)
2008	9.2 ± 10.4	7 (1;63)	1.8 ± 4.3	0 (0;18)	22.7 ± 28.1	9.5 (0;119)	9.8 ± 55.4	0 (0;363)	9.2 ± 10.4	49.5 (0;228)	1.8 ± 4.3	6 (0;22)	22.7 ± 28.1	3 (0;22)	9.8 ± 55.4	15 (0;22)
2009	12.0 ± 25.4	6 (0;164)	7.9 ± 29.7	0 (0;157)	67.2±101	9 (0;353)	0.1±0.4	0 (0; 2)	12.0 ± 25.4	47 (0;224)	7.9 ± 29.7	5,5 (1;23)	67.2 ± 101	5 (1;23)	0.1 ± 0.4	16 (1;23)
2010	9.9 ± 16.4	6 (0;89)	5.0 ± 14.3	0 (0;78)	61.9 ± 80.5	35 (0;348)	0.0 ± 0.3	0 (0;2)	9.9 ± 16.4	41 (0;275)	5.0 ± 14.3	5 (1;37)	61.9 ± 80.5	6 (1;37)	0.0 ± 0.3	15 (1;37)
2011	9.9 ± 42.6	6 (1;362)	9.3 ± 52.6	0 (0;354)	14.7 ± 21.9	6 (0;98)	0.3 ± 2.1	0 (0;18)	9.9 ± 42.6	27 (0;152)	9.3 ± 52.6	5 (1;29)	14.7 ± 21.9	6 (1;29)	0.3 ± 2.1	15 (1;29)
2012	9.9 ± 12.2	5 (0;86)	2.2 ± 5.5	0 (0;24)	13.7 ± 29.6	3 (0;144)	0.5 ± 3.6	0 (0;32)	9.9 ± 12.2	24.5 (0;274)	2.2 ± 5.5	5 (0;37)	13.7±29.6	14 (0;37)	0.5 ± 3.6	16 (0;37)
2013	9.9 ± 5.7	5 (0;31)	2.0 ± 4.8	0 (0;27)	17.0 ± 40.9	6 (0;294)	1.5 ± 8.0	0 (0;58)	9.9 ± 5.7	31 (0;293)	2.0 ± 4.8	5 (1;24)	17.0 ± 40.9	4 (1;24)	1.5 ± 8.0	15 (1;24)
2014	9.9 ± 8.9	6 (0;57)	1.9 ± 4.4	0 (0;22)	14.5 ± 33.7	4 (0;278)	0.0 ± 0.3	0 (0;3)	9.9 ± 8.9	22.5 (0;142)	1.9 ± 4.4	5 (1;50)	14.5 ± 33.7	4 (1;50)	0.0 ± 0.3	15 (1;50)
2015	9.9 ± 5.8	6 (0;42)	11.5 ± 75.1	0 (0;577)	12.5 ± 20.9	5 (0;111)	0.8 ± 4.5	0 (0;39)	9.9 ± 5.8	33 (0;262)	11.5 ± 75.1	5 (0;26)	12.5 ± 20.9	4 (0;26)	0.8 ± 4.5	17 (0;26)
2016	9.9 ± 5.7	6 (0;42)	1.5 ± 5.9	0 (0;37)	12.3 ± 16.6	6 (0;80)	0.0 ± 0.2	0 (0;2)	9.9 ± 5.7	34 (1;83)	1.5 ± 5.9	6 (1;18)	12.3 ± 16.6	4 (1;18)	0.0 ± 0.2	16 (1;18)
2017	9.9 ± 13.6	5 (0;108)	1.2 ± 3.7	0 (0;22)	24.8 ± 40.6	12 (0;256)	0.3 ± 2.6	0 (0;22)	9.9 ± 13.6	37 (0;256)	1.2 ± 3.7	5 (1;15)	24.8 ± 40.6	5 (1;15)	0.3 ± 2.6	17 (1;15)

Legend: sd = standard deviation; Max = maximum value; Min = minimum value.

**Figure 2:**
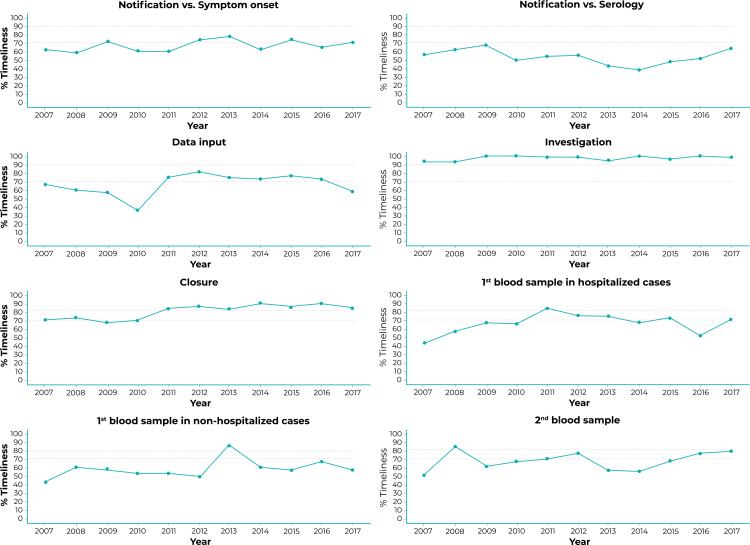
Percentage timeliness of notification in relation to onset of first symptoms, data input, investigation, closure and serological sample collection, São Paulo State, 2007-2017

Notes: Parameters for classifying completeness and timeliness: good (≥ 90.0%), regular (≥ 70.0% to < 90.0%) or poor (if < 70.0%) for all variables except timeliness of “Closure”.

The time trend analyses are shown in Figure 2 and Table 2. Only the “Discharge date” and “Date of second serological sample” completeness curves and the “Closure” timeliness curve had a rising time trend. The trend was stable for all the other variables.

**Table 2:** Time trend analysis of completeness and timeliness of 12 fields of the spotted fever investigation form, São Paulo State, 2007-2017

Study variables	APC^a^	95%CI^b^	p-value	Trend
Completeness				
Date of hospital admission	0.07	-1.02;1.17	0.903	Stable
Date of discharge	5.59	1.19;10.19	0.034	Rising
Date of 1^st^ serological sample	0.04	-1.02;1.11	0.947	Stable
Date of 2^nd^ serological sample	5.35	3.37;7.36	< 0.001	Rising
Timeliness				
From first symptoms up until notification	3.07	0.17;6.05	0.767	Stable
From notification to 1^st^ serological sample	-1.22	-8.71;6.88	0.067	Stable
From notification to data input	2.43	-3.65;8.90	0.461	Stable
From notification to investigation	0.80	-0.30;1.90	0.189	Stable
From notification to closure	4.84	2.46;7.28	0.003	Rising
From first symptoms to 1^st^ serological sample - hospitalized cases	3.47	-2.67;10.00	0.302	Stable
From first symptoms to 1^st^ serological sample - non-hospitalized cases	2.91	-0.63;6.58	0.143	Stable
From 1^st^ serological sample to 2^nd^ serological sample	1.51	-2.62;5.81	0.498	Stable

a) APC = Annual Percentage Change; b) 95%CI: 95% confidence interval.

Parameters for classifying completeness and timeliness of “Serologic analyses”: good (≥ 80.0%); regular (70.0% to < 80.0%) and poor (< 70.0%).

Although its trends remained stable over the period, “investigation” had good timeliness in 2009, 2010, 2014 and 2016; while “closure’ had good timeliness with effect from 2011. The same was not found for serological sampling and other timeliness variables.

**Figure 3:**
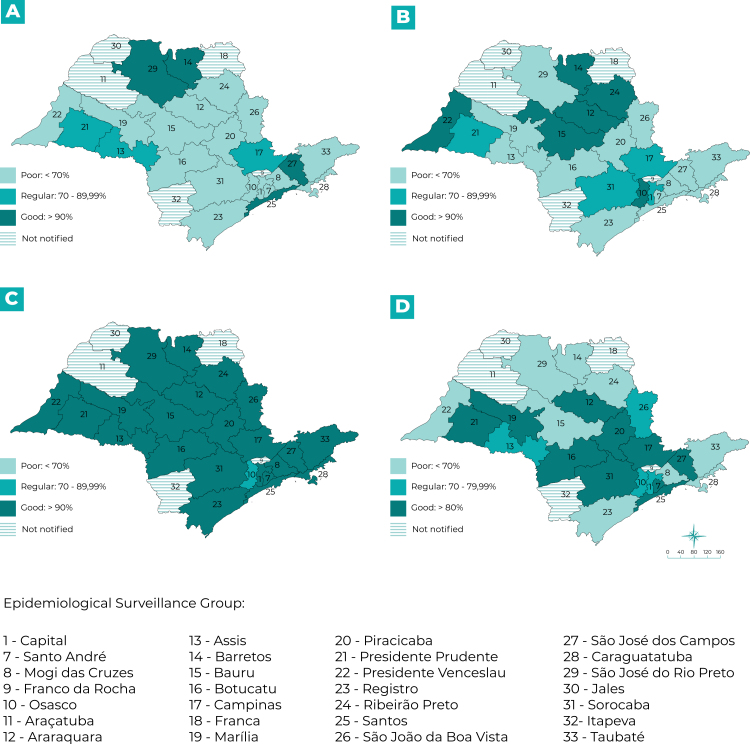
Evaluation of percentage timeliness per Epidemiological Surveillance Group for the “A - Notification”, “B - Data input”, “C - Investigation”, and “D - Closure” variables of the spotted fever investigation forms, São Paulo State, 2007-2017

Ninety-seven municipalities distributed over 23 Epidemiological Surveillance Groups (Grupos de Vigilância Epidemiológica - GVE) notified confirmed cases of SF; the exceptions were the Araçatuba, Franca, Franco da Rocha, Itapeva and Jales GVEs, where no cases were notified in the period studied. In our analysis by GVE, we examined timeliness of the “Notification”, “Data input”, “Investigation” and “Closure” fields, as shown in Figure 3. With regard to “Notification” (Figure 3A), only four GVEs had good timeliness: Barretos, Santos, São José dos Campos and São José do Rio Preto. In the case of “Data input” (Figure 3B), six GVEs had good timeliness: Osasco, Araraquara, Barretos, Bauru, Presidente Venceslau and Ribeirão Preto. In relation to “Investigation” (Figure 3C), all the GVEs had good timeliness; except for the Osasco GVE, for which “Investigation” was classified as regular. Ten GVEs achieved good classification for “Closure” (Figure 3D).

## DISCUSSION

The main finding of this study is that in the period from 2007 to 2017, the completeness of notifications of confirmed cases of SF was considered good^14^ for most of the variables studied; however, the timeliness of laboratory “Investigation” could not be considered adequate.^14^ In 2017, the municipalities where confirmed cases of SF were reported concentrated almost 60% of the state’s population, this being a relevant finding.

The timeliness of SF case “Investigation” was considered good in São Paulo State in the period studied. This could result from adequate health surveillance actions, with identification of probable infection sites for this disease, detailed in São Paulo Epidemiological Bulletins.^23^ ^),(^ ^24^ The good completeness found for most variables, in more than half of the years studied, corroborates this result.

Another satisfactory finding of the study was the rising trend in completeness observed in two of the post-notification follow-up fields, “Date of discharge” from hospital and “Date of collection of second serological sample”, as well as a rising trend in the timeliness of “Closure”. The absence of a falling trend for timeliness in the other analyses is also relevant.

In the period 2007-2017, the increase in the completeness of the essential fields suggests improvement in the quality of information and knowledge about the disease. This may be the result of the Epidemiological Surveillance Service actively tracing notified cases to complete the information, reinforced by the obligation to immediately notify SF with effect from 2014.^9^ The problem is relevant, because in Brazil, shortcomings in data filling have been reported in other studies on different notifiable diseases.^13^ ^),(^ ^25^ ^),(^ ^26^

Several factors can interfere with the filling in of notification forms, such as (i) not being aware of the importance of the information collected, (ii) the perception that notification is a merely bureaucratic task, (iii) loss of motivation and work overload among the professionals involved, (iv) definition of other priorities by decision-making bodies, as well as variations according to the characteristics of the local health system.^16^ ^),(^ ^17^ ^),(^ ^27^

Timely case investigation means that related actions were also undertaken, also in a timely manner, possibly including environmental research to identify the vector tick species and probable infection site.^7^ The fact that almost all cases were investigated in a timely manner indicates that the Epidemiological Surveillance Service operated efficiently in most of the state during the entire period, contributing to the accuracy of knowledge of the epidemiological profile of the disease in the state and the distribution of the vector tick.

Good timeliness of suspected diagnosis is fundamental for guiding early correct antibiotic treatment, which can reduce the case fatality ratio.^6^ Correct classification of the risk of disease occurring in the territory is also important, in order to support medical decisions.

The study showed that the timing of sample collection for serological analysis was a parameter that needs to be improved. This is essential information for classifying cases and, consequently, probable infection sites and areas of transmission.^24^ Missing or inadequate laboratory information on the SINAN, or even its untimely input to the system, can lead to inaccurate results and jeopardize epidemiological investigation of cases.^7^ ^),(^ ^14^ ^),(^ ^23^ ^),(^ ^24^ Integration between SINAN databases and reference laboratory databases is recommended in order to get around this limitation, as this would result in better data quality.

The prolonged time lapse found between onset of symptoms and notification may be associated with the person’s delay in seeking medical attention or may signal a delay in suspected diagnosis, not necessarily meaning a failure in the notification system.^19^ ^),(^ ^26^ This delay can interfere in case outcomes, as well as interfering in actions to control disease and prevent infection risks in humans.^14^ ^),(^ ^24^

More than 80.0% of the cases were closed in a timely manner, providing a good evaluation of the epidemiological profile and, consequently, implementation of effective control and prevention measures to be adopted by the surveillance services.^28^ It should be added that in this period, there was very close contact with the state surveillance system, according to which, over time, specific integrated actions were implemented between the central level of epidemiological surveillance and local health services to achieve the best approach to the disease. This may have been reflected in the good “Case closure” results found with effect from 2011.

Regarding spatial distribution, the identification of five GVEs with no notifications is noteworthy. This situation needs specific investigation, because the presence of vector ticks has already been described in the entire state of São Paulo^3^ ^)-(^ ^5^ ^),(^ ^10^ ^),(^ ^29^ ^),(^ ^30^ as have hosts that spread the etiologic agent (dogs and capybaras).^3^ ^),(^ ^4^ ^),(^ ^6^ ^),(^ ^10^ ^),(^ ^23^ ^),(^ ^24^ ^)^ The fact that “Notification” timeliness was found to be inadequate in more than half of the territories suggests the need for communication campaigns about SF directed to the population at risk, in addition to frequent training of health professionals.

“Investigation” timeliness was good in almost all territories in which notifications were made, suggesting the correlation of actions in a timely manner, starting with case recording. However, the timeliness of “Closure” and “Data input”, commonly considered to be bureaucratic activities, are unsatisfactory in most of São Paulo State, which may imply the need to reassess the management of activities inherent to the process.

As limitations of this study, using a database that only contained confirmed cases did not allow us to analyze the quality of the database as a whole, including suspected and discarded cases. Moreover, it was not possible to resolve all the inconsistencies of all the cases made available - despite repeated efforts - although this only resulted in the exclusion of a tiny part (< 5%) of the initial database, with no impact on the results found.

This study allowed us to gain knowledge of the profile of SF data quality, providing information for public health authorities and their situation analysis of the disease over time, this being reflected in the related health indicators. As the first regionalized evaluation of these parameters in São Paulo state, this study contributes to the improvement of data collection about SF in the cities comprising the state’s different GVEs. This allows better allocation of resources in surveillance areas, health professional training and adoption of health policies by the state’s regions.

To date, this is the only study known to have evaluated completeness and different parameters of timeliness for data on SF held on the SINAN, including their spatial distribution. Finally, it should be emphasized that the analysis of the time lapses between serological sample collections from confirmed cases was an original initiative of this research.

## References

[1] Sevá AP, Martins TF, Munõz-Leal S, Rodrigues AC, Pinter A, Luz HR (2019). A human case of spotted fever caused by Rickettsia parkeri strain Atlantic rainforest and its association to the tick Amblyomma ovale. Parasit Vectors.

[2] Ribeiro CM, Costa VM, Carvalho JLB, Mendes RG, Bastos PAS, Katagiri S (2020). Brazilian spotted fever: a spatial analysis of human cases and vectors in the state of São Paulo, Brazil. Zoonoses Public Health.

[3] Oliveira SV, Guimarães JN, Reckziegel GC, Neves BMC, Araújo-Vilges KM, Fonseca LX (2016). An update on the epidemiological situation of spotted fever in Brazil. J Venom Anim Toxins Incl Trop Dis.

[4] Polo G, Acosta CM, Labruna MB, Ferreira F, Brockmann D (2018). Hosts mobility and spatial spread of Rickettsia rickettsii. PLoS Comput Biol.

[5] Binder LC, Ramírez-Hernández A, Serpa MCA, Moraes-Filho J, Pinter A, Scinachi CA (2021). Domestic dogs as amplifying hosts of Rickettsia rickettsii for Amblyomma aureolatum ticks. Ticks Tick Borne Dis.

[6] Araújo RP, Navarro MBMA, Cardoso TAO (2015). Febre maculosa no Brasil: estudo da mortalidade para a vigilância epidemiológica. Cad Saude Colet.

[7] Ministério da Saúde (BR). Secretaria de Vigilância em Saúde. Coordenação-Geral de Desenvolvimento da Epidemiologia em Serviços (2019). Guia de Vigilância em Saúde.

[8] Brasil. Ministério da Saúde (2001). Portaria nº 1943, de 18 de outubro de 2001. Define a relação de doenças de notificação compulsória para todo território nacional.

[9] Brasil. Ministério da Saúde (2014). Portaria no 1.271, de 6 de junho de 2014. Define a Lista Nacional de Notificação Compulsória de doenças, agravos e eventos de saúde pública nos serviços de saúde públicos e privados em todo o território nacional, nos termos do anexo, e dá outras providências.

[10] Oliveira SV., Willemann MCA, Gazeta GS, Angerami RN, Gurgel-Gonçalves R (2017). Predictive factors for fatal tick-borne spotted fever in Brazil. Zoonoses Public Health.

[11] Ministério da Saúde (BR). Departamento de Informática do SUS - Datasus. Informações de Saúde (TABNET) (2022). Epidemiológicas e Morbidade. Febre Maculosa - Casos confirmados notificados no Sistema de Informação de Agravos de Notificação - Brasil.

[12] Secretaria de Saúde do Estado (SP). Centro de Vigilância Epidemiológica “Prof Alexandre Vranjac” (2022). Dados estatísticos - febre maculosa.

[13] Marques CA, Siqueira MM, Portugal FB (2020). Assessment of the lack of completeness of compulsory dengue fever notifications registered by a small municipality in Brazil. Cien Saude Colet.

[14] Ministério da Saúde (BR). Secretaria de Vigilância em Saúde (2005). Guia de vigilância epidemiológica.

[15] German RR, Lee LM, Horan JM, Milstein RL, Pertowski CA, Waller MN (2001). Updated guidelines for evaluating public health surveillance systems: recommendations from the GuidelinesWorking Group. Recommendations and Reports.

[16] Siqueira PC, Maciel ELN, Catão RC, Brioschi AP, Silva TCC, Prado TN (2020). Completude das fichas de notificação de febre amarela no estado do Espírito Santo, 2017. Epidemiol Serv Saude.

[17] Canto VB, Nedel FB (2020). Completude dos registros de tuberculose no Sistema de Informação de Agravos de Notificação (Sinan) em Santa Catarina, Brasil, 2007-2016. Epidemiol Serv Saude.

[18] Goto DYN, Larocca LM, Felix JVC, Kobayashi VL, Chaves MMN (2016). Assessment of the timeliness for notification of dengue in the state of Paraná. Acta Paul Enferm.

[19] Swaan C, van den Broek A, Kretzschmar M, Richardus JH (2018). Timeliness of notification systems for infectious diseases: a systematic literature review. PLoS One.

[20] Instituto Brasileiro de Geografia e Estatística (2017). Cidades e estados - São Paulo.

[21] Antunes JL, Cardoso MR (2015). Uso da análise de séries temporais em estudos epidemiológicos. Epidemiologia e Serviços de Saúde.

[22] São Paulo (Estado) (1995). Decreto no 40.083, de 15 de maio de 1995. Organiza as Direções Regionais de Saúde, extingue 41 (quarenta e um) Escritórios Regionais de Saúde e dá providências correlatas.

[23] Pinter A, Sabbo C, Leite R, Spinola R, Angerami R (2021). Inf técnico sobre Febre Maculosa. Bol Epidemiol Paul.

[24] Pinter A, Costa CS, Holcman MM, Camara M, Leite RM (2016). A febre maculosa brasileira na região metropolitana de São Paulo. Bol Epidemiol Paul.

[25] Oliveira MEP, Soares MRAL, Costa MCN, Mota ELA (2009). Avaliação da completitude dos registros de febre tifóide notificados no Sinan pela Bahia. Epidemiol Serv Saude.

[26] Chehab MA, Bala MO, Al-Dahshan A, Selim NA, Al-Romaihi HE, Al-Thani M (2018). Evaluation of the completeness and timeliness of national malaria surveillance system in Qatar, 2016. Cureus.

[27] Rocha MS, Bartholomay P, Cavalcante MV, Medeiros FC, Codenotti SB, Pelissari DM (2020). Sistema de Informação de Agravos de Notificação (SINAN): principais características da notificação e da análise de dados relacionada à tuberculose. Epidemiol Serv Saude.

[28] Brasil. Ministério da Saúde (2005). Instrução normativa nº 02/SVS/MS, de 22 de novembro de 2005. Regulamenta as atividades da vigilância epidemiológica com relação à coleta, fluxo e a periodicidade de envio de dados da notificação compulsória de doenças por meio do Sistema de Informação de Agravos de Notificação - SINAN.

[29] Luz HR, Costa FB, Benatti HR, Ramos VN, Serpa MCA, Martins TF (2019). Epidemiology of capybara-associated Brazilian spotted fever. PLoS Negl Trop Dis.

[30] Nunes FBP, Silva SC, Cieto AD, Labruna MB (2019). The dynamics of ticks and capybaras in a residential park area in southeastern Brazil: Implications for the risk of Rickettsia rickettsii infection. Vector Borne Zoonotic Dis.

